# The Incidence of Pediatric Tibial Spine Fractures Is Greater and Peaks Later in Male Patients

**DOI:** 10.1016/j.asmr.2021.12.005

**Published:** 2022-01-21

**Authors:** Christopher J. DeFrancesco, Alexandra Tananbaum, Drake G. LeBrun, Peter D. Fabricant

**Affiliations:** Hospital for Special Surgery, New York, New York, U.S.A.

## Abstract

**Purpose:**

To use government-curated databases to produce incidence estimates for pediatric tibial spine fractures (TSFs) by age and sex. This study also describes the relative frequency of operative versus nonoperative management for TSFs by age and sex.

**Methods:**

US Healthcare Cost and Utilization Project databases were used to identify cases of TSF among patients aged 7 to 18 years in the year 2016. Patient-linked deidentified data from New York, Maryland, and Florida were gathered from state databases, and repeat visits by the same patient were collapsed into individual records. TSF incidence was then calculated, with U.S. census data used to determine the number of children at risk. The proportion of cases treated nonoperatively was determined based upon procedural codes.

**Results:**

In New York, Florida, and Maryland, 185 cases of TSF were found. Male patients accounted for 69.7% of cases. Incidence peaked at 9.3 per 100,000 at age 14 years for male patients and at 3.4 per 100,000 at age 9 years for female patients. In total, 57.9% of TSF cases were treated nonoperatively. The overall incidence of TSF was 2.8 cases per 100,000 for people aged 7 to 18 years.

**Conclusions:**

This study confirms a difference in incidence by sex for pediatric TSFs, with male patients having a greater peak incidence that also occurs at an older age. Most cases in this study were treated nonoperatively.

**Clinical Relevance:**

Due to the relative infrequency of TSFs in the pediatric population, there is a limited understanding of the epidemiology and treatment of these fractures. The use of data from a large patient database may provide valuable epidemiologic information about this uncommon injury.

While tibial spine fractures (TSFs) are a well-described knee injury in the pediatric population, existing studies on the topic are mainly small single-center series. As a result, there is a limited understanding of the epidemiology of these injuries in the larger population. Database studies may provide an effective approach for compiling a critical number of cases from which to estimate incidence and describe trends in treatments and outcomes.

A recent study identified more than 800 cases of TSFs from a database of privately insured patients and showed that the injury was more common in male than female patients. The authors also showed that case burden in female patients peaked earlier than in male patients.^1^ However, this previous study could not estimate the per-capita incidence of TSFs due to the lack of definable numbers of individuals at risk in the database used. Furthermore, that study’s conclusions regarding TSF case burden and the frequency of common treatment options have not yet been corroborated.

Injury incidence estimates are among the most common and helpful measures of disease burden, because they allow clinicians, epidemiologists, and health systems to better understand the general risk of injury in a specific population. Thus, the purposes of this study were to estimate the incidence of pediatric TSFs by age and sex and to determine the relative frequency of operative versus nonoperative management for TSFs by age and sex. It was hypothesized that incidence would be greater in male patients and that nonoperative management would predominate.

## Methods

Institutional review board approval was not required for this retrospective study of deidentified data.

### Data Sources

The Healthcare Cost and Utilization Project (HCUP) includes a family of national and state health care databases that compile encounter-level health care data. HCUP databases have been used extensively in previously published medical research studies.^2^, ^3^, ^4^, ^5^ Participating states publish 3 different databases yearly: the State Inpatient Databases (SID), State Emergency Department Databases (SEDD), and State Ambulatory Surgery and Services Databases (SASD). Each state’s SID contains information on inpatient hospital discharges, including emergency department visits that led to hospitalizations. The SID^6^ is thought to capture >95% of all hospital discharges. In contrast, the SEDD^7^ contains data for all hospital-affiliated emergency department visits that did not lead to hospitalizations. Lastly, the SASD^8^ includes data for outpatient services (including ambulatory surgery) at hospital-owned facilities. While database information is deidentified, some states include a VisitLink variable, which can be used to link visits across databases attributable to the same patient.

For 2016, 33 U.S. states published HCUP state databases, whereas only 16 states included the VisitLink variable. Understanding the importance of using this linking variable to eliminate repeat visits and thus provide a reliable estimate of disease, the authors chose to use state databases from this list of 16. Among these states, databases from New York, Florida, and Maryland were used because they were among the most populous states available. Less-populous states were not included due to the cost of additional databases with respect to the expected number of cases they would yield. Understanding that the *International Classification of Diseases, Tenth Revision* (ICD-10), created a specific diagnosis code for TSF that previously did not exist, the year 2016 was chosen for the analysis. This was the first year that the SEDD and SASD reported ICD-10 codes and the most recent year with data available for all 3 chosen states.

### Case Identification

#### Noninpatient Databases (SEDD and SASD)

The authors used ICD-10 and Current Procedural Terminology (CPT) codes to find cases of tibial spine fractures from the SEDD and SASD. This was done according to Table 1. To avoid misclassification, any subject with the diagnosis code for tibial tubercle fracture (ICD-10 code S82.15) was excluded. This was done because, in previous ICD editions, tibial spine fractures and tibial tubercle fractures shared the same diagnosis code.

**Table 1:** TSF Case Identification from the SEDD and SASD

Subgroup	Group Title	Criteria
A_1_	TSF	ICD S82.11
B_1_	TSF treated surgically	A_1_ and (CPT 29888 or 2985∗ or 27540)
C_1_	TSF treated nonoperatively	A_1_ and (not B_1_)

NOTE. ICD codes are ICD 10 diagnosis codes. ICD S82.15 (tibial tubercle fracture) was excluded.

CPT, Current Procedural Terminology; ICD, *International Classification of Diseases*; SASD, State Ambulatory Surgery and Services Databases; SEDD, State Emergency Department Databases; TSF, tibial spine fracture.

∗ 2985 includes codes 29850, 29851, 29855, and 29856.

#### Inpatient Database (SID)

The 2016 SID does not use CPT codes to denote procedures. Rather, it uses ICD-10-PCS (ICD procedure) codes. With this in mind, subgroup assignment was somewhat different for these databases, proceeding as outlined in Table 2. In these databases, the ICD-10-PCS (procedure) codes for surgery on the “Bursae and Ligaments” or “Lower Bones” was considered to represent surgical treatment for a TSF fracture (see subgroup B_2_ in Table 2).

**Table 2:** TSF Case Identification From the SID

Subgroup	Group Title	Criteria
A_2_	TSF	ICD S82.11
B_2_	TSF treated surgically	A_2_ and (ICD-10-PCS code 0M∗ or ICD-10-PCS code 0Q∗)
C_2_	TSF treated nonoperatively	A_2_ and (not B_2_)

NOTE. ICD codes are ICD 10 diagnosis codes. ICD S82.15 (tibial tubercle fracture) was excluded.

ICD, *International Classification of Diseases*; PCS, Procedure Coding System; SID, State Inpatient Database; TSF, tibial spine fracture.

∗ Denotes wildcard character.

### Analysis

#### State Databases

Subgroups were defined based on variables as described previously, with Group A (A_1_ from Table 1 and A_2_ from Table 2) representing all patients with TSFs, Group B (B_1_ Table 1 and B_2_ from Table 2) representing all patients with a TSF treated surgically, and Group C (C_1_ and C_2_ together) representing patients with a TSF treated nonoperatively. Repeat visits were manually eliminated based upon duplicate VisitLink variables. When multiple entries with the same VisitLink variable included a code for surgery, the collapsed final entry for the patient preserved the surgery-associated variables rather than those associated with nonsurgical visits. In this way, it was assured that each entry in the final dataset had a unique VisitLink variable, indicating that each represented a unique individual with a TSF rather than a single episode of care.

Descriptive statistics, including the proportion of patients treated nonoperatively, were compiled from the final dataset. Cases of TSF were separated by sex for patients 7 to 18 years old. Subjects were further divided by age in 2-year increments and by sex to form 12 sex/age strata. The overall number of people at-risk in New York, Florida, and Maryland in 2016 was defined by sex and age using the U.S. census online data tool.^9^ Incidence was then calculated using the number of cases for the 3 states combined divided by the total number of individuals at-risk in those states; these values were calculated separately for each sex/age stratum.

### Statistical Testing

Database analysis was performed using Stata, version 14.2 (StataCorp, College Station, TX). Data were then exported to Microsoft Excel (Microsoft, Redmond, WA) for further analysis. Descriptive statistics were performed within Excel. Statistical tests, including χ^2^ tests, were performed in Stata. The threshold for statistical significance was set at *P* ≤ .05, and all comparisons were 2-tailed.

## Results

### Incidence

The databases revealed 185 cases of TSF in New York, Florida, and Maryland among patients 7 to 18 years old in 2016, translating to an incidence of 2.8 cases per 100,000 individuals in that age range. Most TSFs were among male patients (n = 129, 69.7%). The most common age for male patients to have a TSF was age 13 to 14 years (36.4% of male cases). For female patients, the most common age was 9 to 10 years (30.4% of female cases).

**Fig 1:**
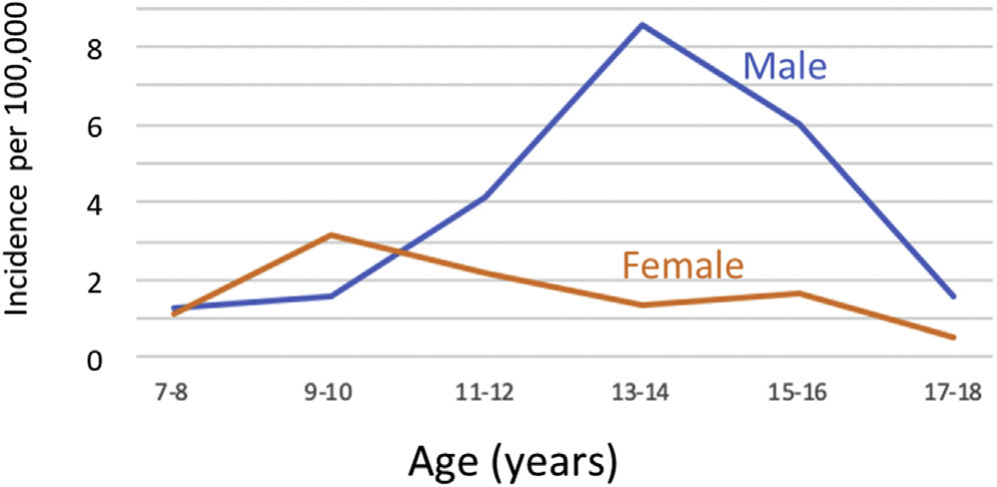
Incidence of tibial spine fractures (TSFs) from ages 7 to 18 years in male and female patients. Incidence for female patients may plateau over the ages of 9-12 years.

The incidence of TSFs peaked for male patients at age 14 (9.3 per 100,000). For female patients, incidence peaked at a lower magnitude and an earlier age (3.4 per 100,000 at age 9). This incidence was nearly equaled at age 12 years (3.3 per 100,000), suggesting that the incidence for female patients may plateau over the ages of 9 to 12 years. Incidence by age and sex is further illustrated in Figure 1 and Table 3. It should be noted that the HCUP stipulates that, when using their databases, no cell values <10 may be reported. As such, Table 3 includes several cells marked as “<10.” It should be made clear that the actual numbers for these cells were known locally during data analysis, allowing for proper summation of cases and incidence calculations.

**Table 3:** Incidence of TSFs in New York, Florida, and Maryland in 2016 Stratified by Age and Sex

	Age, y					
	7-8	9-10	11-12	13-14	15-16	17-18
Male						
Case number, n∗	<10	<10	22	47	34	<10
At-risk†	Censored	Censored	534,218	547,247	581,039	Censored
Incidence	1.27	1.58	4.12	8.59	5.85	1.55
Female						
Case number, n∗	<10	17	12	<10	<10	<10
At-risk†	Censored	534,334	546,462	Censored	Censored	Censored
Incidence	1.15	3.18	2.20	1.36	1.64	0.53

NOTE. Incidence is reported as cases per 100,000 persons.

HCUP, Healthcare Cost and Utilization Project; TSF, tibial spine fracture.

∗ HCUP requires that any values less than 10 be censored.

† In any column in which the case number is <10, the number at-risk (although known for analysis) is not published. This is to prevent back-calculation of the number at risk based on the reported incidence.

### Treatment

Most cases of TSF were treated nonoperatively (57.9%). The rate of nonoperative treatment varied by state (47.0%-65.6%), although this difference did not reach statistical significance (*P* = .064). Male and female patients underwent surgical treatment at similar rates (41.1% vs 44.4%, respectively; *P* = .500). The rate of nonoperative treatment varied by 2-year age division, ranging from 46.2% to 64.3%; this did not reach statistical significance (*P* = .489). Although the rate of nonoperative treatment varied by sex/age strata (range 33.3%-71.4%), this did not reach statistical significance (*P* = .187).

## Discussion

The current study showed that, among pediatric patients, TSF cases were more common among males versus females peaked later in males than in females and were more likely to be treated nonoperatively in both sexes. While a recent study reported that TSF case burden is greater in young male patients and that case burden among male patients peaks later than in female patients,^1^ incidence estimates could not be calculated in that study due to the lack of a known at-risk population. Thus, the per capita annual TSF incidence figures presented in the current study are the first such estimates published in the medical literature.

In contrast to the current study’s report that the incidence of TSFs among patients aged 7 to 18 years was less than 3 per 100,000 in the year 2016, a recent study estimated the risk of ACL rupture in patients younger than 18 years of age to be much greater, at more than 23 per 100,000 person-years.^10^ The reasons behind the difference in incidence between these two similar but distinct injuries are unclear but may have to do with epiphyseal maturation, variable exposure to certain high-risk activities, or differences in diagnostic sensitivity. The authors believe that biological phenomena regarding bone maturation and variations in risk exposure also may be responsible for differences in TSF incidence by age and sex noted in the current study. For example, it is possible that—due to changes in molecular makeup—the maturing epiphysis is most susceptible to tibial spine avulsion injury approximately 5 years before physeal closure. Seeing as the proximal tibial physis tends to close about 2 years earlier in female than in male patients, this would account for why the incidence for females peaks a few years before the incidence for males peaks. In contrast, it is possible that differential exposure to high-risk activities (like American-style football) explains why male patients tend to account for more than twice as many TSF cases as female patients. With these theories in mind, it should be pointed out that further basic science and larger clinical studies will be needed to determine the true causes for the aforementioned differences.

Unfortunately, such large clinical studies are not easily completed, primarily due to the rarity of pediatric TSFs. Considering that there are likely somewhere between 1,000 and 2,000 pediatric TSFs in the United States each year (based on extrapolation from our data), compared with more than 1,400 members of the Pediatric Orthopaedic Society of North America,^11^ it can be deduced that few pediatric orthopaedic surgeons will see more than one pediatric TSF in a given year. The low volume of these injuries helps to explain why most studies on the subject are retrospective series compiled over several years. The study at hand attempted to overcome these challenges through the use of databases that compile information from multiple centers.

In comparison with the current study, a retrospective series of 122 pediatric TSF cases treated over an 18-year period at a single center was recently compiled by Axibal et al.^12^ In their series, 24% of cases were caused by bicycle accidents and 20% were caused by American-style football injuries. The authors noted that younger age was associated with more severe fracture displacement, but they did not detail what proportion of patients underwent surgical intervention. The male patients in this series had a mean age of 11.6 years at the time of injury, compared with an average age of 9.8 years at the time of injury for female patients. Meanwhile, the current study similarly showed a difference in typical age of injury for male and female patients, with incidence for male patients peaking around 14 years of age and the incidence for female patients plateauing over ages 9 to 12 years. In addition, the series by Axibal et al.^12^ exhibited a male:female case ratio of 2.2:1, consistent with the 2.3:1 ratio seen in the current study and the 2.2:1 ratio seen in another recent study.^1^

While the previous work by Axibal et al. did not detail the proportion of cases treated nonoperatively, another recent series reported that only one third of patients with displaced TSFs were treated nonoperatively.^13^ In contrast, more than one-half of cases were treated nonoperatively in the current study. However, any interpretation of this reported rate must be done cautiously in the absence of more detailed knowledge of each case (i.e., fracture displacement and fragmentation), as it is likely that the greater rate of closed treatment observed is at least partly due to the inclusion of nondisplaced TSFs appropriately treated nonoperatively. In the end, it may be interesting to note that more TSF cases were treated nonoperatively than operatively, but it was not possible to determine whether these treatment strategies were appropriate in the absence of granular case data.

### Limitations

The current study has several limitations. First, the type of surgery done in cases treated operatively could not be reliably established in this study. While the procedural codes used were reliable for classifying subjects as surgically or nonoperatively treated, they could not be used to decipher between suture-only fixation, screw fixation, anterior cruciate ligament repair, or anterior cruciate ligament reconstruction. As noted previously, the databases used also lacked detailed case data like fracture displacement, Meyers and McKeever classification, surgeon training, physical therapy treatments, and more; this prevented any comparisons between treatment outcomes along with other higher-level comparisons.

In addition, statistical comparisons made by sex, state, or age in the current study (see the Treatment paragraph in the Results section) must be interpreted cautiously, as the study was not powered for these appraisals, creating the possibility for type II error in these comparisons. Although case numbers could have been boosted by using more state databases, this was limited by data availability and the cost of database acquisition. A longitudinal national database including inpatient and outpatient visits for young patients would have been ideal to maximize case numbers and generalizability for this study, but, unfortunately, the authors are not aware of any such resource.

Misclassification must always be considered in any database study, as the correct definition of cases relies on proper coding in the database. In this study, there also may have been some misclassification events related to year turnover. For instance, some patients with follow-up appointments in 2016 could have been included in our dataset, even though their injuries were actually in 2015. In contrast, some patients with injuries in 2016 may have been included in our dataset as nonoperative cases, even though they had surgery in the beginning of 2017 (and thus surgical data was not available in the 2016 databases). The inpatient databases (SID) also used a different procedure coding system than the other databases (ICD-PCS codes rather than CPT codes, see Tables 1 and 2), presenting another potential source of misclassification in this study.

Lastly, the authors recognize that the HCUP databases are not 100% comprehensive; there may be a small proportion of care that goes uncaptured, contributing to an underestimation of incidence. The authors believe this risk to be small, as the proportion of uncaptured cases is not large. In addition, most patients with a TSF would be expected to have >1 episode of care, decreasing the chance that any one of them would avoid being captured in an HCUP database at some point during their care. There also is no reason to believe that missing data would be unevenly distributed by age or sex, suggesting that the sex differences in incidence are likely real even if there is some influence of misclassification.

## Conclusions

This study confirms a difference in incidence by sex for pediatric TSFs, with male patients having a greater peak incidence that also occurs at an older age. Most cases in this study were treated nonoperatively.

## Supplementary Data

ICMJE author disclosure forms
